# Evaluation of the Effects of *Vaccinium arctostaphylos* L. Fruit Extract on Serum Lipids and hs-CRP Levels and Oxidative Stress in Adult Patients with Hyperlipidemia: A Randomized, Double-Blind, Placebo-Controlled Clinical Trial

**DOI:** 10.1155/2014/217451

**Published:** 2014-01-23

**Authors:** Rasool Soltani, Mustafa Hakimi, Sedigheh Asgary, Syed Mustafa Ghanadian, Mahtab Keshvari, Nizal Sarrafzadegan

**Affiliations:** ^1^Department of Clinical Pharmacy and Pharmacy Practice, Faculty of Pharmacy and Pharmaceutical Sciences, Isfahan University of Medical Sciences, Isfahan, Iran; ^2^Isfahan Cardiovascular Research Center, Isfahan Cardiovascular Research Institute, Isfahan University of Medical Sciences, Isfahan, Iran; ^3^Department of Pharmacognosy, Faculty of Pharmacy and Pharmaceutical Sciences, Isfahan University of Medical Sciences, Isfahan, Iran

## Abstract

*Background.* Dyslipidemia produces atherosclerosis, which in turn results in coronary artery disease (CAD). Atherosclerosis is being considered as an inflammatory disease. *Vaccinium arctostaphylos *L. is a plant with fruits rich in anthocyanins. The aim of this study was to evaluate the effects of fruit extract of this plant on serum levels of lipids, hs-CRP, and malondialdehyde (MDA) as a marker of oxidative stress, in hyperlipidemic adult patients. *Methods.* In this randomized, double-blind, placebo-controlled clinical trial, 50 hyperlipidemic adult patients were randomly and equally assigned to receive either medicinal (*V. arctostaphylos* fruit extract) or placebo capsules twice daily for 4 weeks. Each medicinal capsule contained 45 ± 2 mg of anthocyanins. Fasting serum levels of total cholesterol, TG, LDL-C, HDL-C, hs-CRP, and MDA were obtained before and after the intervention and compared. *Results. V. arctostaphylos* fruit extract significantly reduced total cholesterol (*P* < 0.001), LDL-C (*P* = 0.004), TG (*P* < 0.001), and MDA (*P* = 0.013) compared to placebo but did not have any significant effect on HDL-C (*P* = 0.631) and hs-CRP (*P* = 0.190). *Conclusion.* Fruit extract of *Vaccinium arctostaphylos* has beneficial effects on serum lipid profile and oxidative stress in hyperlipidemic adult patients. Therefore, it could be considered as a supplement for treatment of dyslipidemia and prevention of atherosclerosis development.

## 1. Introduction

Dyslipidemia (one or more abnormalities of blood lipids) produces atherosclerosis, which in turn produces coronary heart disease (CHD) and coronary artery disease (CAD) including chronic stable angina, unstable angina, myocardial infarction, and ischemic cardiomyopathy [1]. Coronary atherosclerosis is the leading cause of death in developed countries [2]. Therefore, successful management of dyslipidemias alters the natural course of atherosclerosis and reduces CHD. Atherosclerosis is being considered as an inflammatory disease, since inflammatory processes play a key role in different stages of plaque development [3]. These include the activation of endothelial cells (ECs) leading to expression of adhesion molecules that attract inflammatory cells (e.g., neutrophils, T-cells, and monocytes) into the early atherosclerotic lesion. Within the plaque, smooth muscle cells (SMCs) and ECs secrete proinflammatory mediators that stimulate monocyte differentiation into macrophages. These macrophages further develop into foam cells upon uptake of oxLDL and locally amplify the inflammatory response, thereby attracting more immune cells and inducing migration of SMCs into the plaque [4, 5].

High-sensitivity C-reactive protein (hs-CRP), a plasma protein synthesized by the liver, is a sensitive and dynamic systemic marker of inflammation [6]. CRP binds to LDL [7, 8] and is present in atherosclerotic plaques [9], so it has been proposed that CRP may have a causal role in coronary heart disease. A systematic review of 31 published prospective cohort studies has suggested that elevated CRP concentrations were associated consistently with increased CHD risk [10]. Therefore, it is possible that drugs with CRP-lowering property could reduce cardiovascular events in high-risk patients. In JUPITER trial, rosuvastatin, compared to placebo, reduced the combined primary endpoint of MI, stroke, arterial revascularization, hospitalization for unstable angina, or death from cardiovascular causes in patients with below average (<130 mg/dL) levels of LDL-C, and high levels of hs-CRP (>2 mg/dL) [11].

Oxidative stress, which occurs in response to an altered metabolic state, apoptosis, and lipid peroxidation, is additionally involved in the pathogenesis of atherosclerosis [12, 13]. Hyperlipidemia increases the production of reactive oxygen species (ROS) by endothelial cells, smooth muscle cells, and macrophages, leading to oxidative stress induction and low density lipoprotein (LDL) oxidation [13, 14]. The development of atherosclerosis is accompanied by an accumulation of oxidized LDL (oxLDL), one of the major oxidized lipids, in the arterial wall [12]. Therefore, phytochemicals and antioxidants that inhibit the production of ROS might have clinical value for treatment of atherosclerosis [15].

*Vaccinium arctostaphylos *L. (Caucasian Whortleberry) is a plant found in northern forests of Iran and commonly known as “Qaraqat.” The fruits (berries) of this plant are rich in anthocyanins [16]. Anthocyanins are compounds with antioxidant, antiatherosclerotic, and antihyperlipidemic activities [17–21]. The aim of this study was to evaluate the effects of fruit extract of this plant on serum levels of lipids, hs-CRP, and malondialdehyde (MDA) as a marker of oxidative stress, in hyperlipidemic adult patients.

## 2. Materials and Methods

### 2.1. Plant Material and Extraction

Fresh ripe berries of *V. arctostaphylos* were collected from the forests of Asalem in the mountain chains of Alborz in the north of Iran in August 2012 and were identified by the Pharmacognosy Department of the Faculty of Pharmacy, Isfahan University of Medical Sciences, Isfahan, Iran. After drying at room temperature (20–22°C), the berries were extracted by maceration with ethanol 70%. The extract was then filtrated, concentrated under vacuum, and standardized by spectroscopic determination of anthocyanin content.

### 2.2. Standardization of the Extract

Total anthocyanin content was determined by the pH differential method [22]. Experiments were carried out in two replicates and data were expressed as mmol cyanidin-3-glucoside equivalent. Briefly, two samples of 1 g dried extract were mixed with 10 mL of buffer solution with pH = 1 (125 mL of 0.2 M KCl and 375 mL of 0.2 M HCl) and 10 mL of buffer solution with pH = 4.5 (400 mL of 1 M sodium acetate, 240 mL of 1 M HCl, and 360 mL of water). Both solutions were diluted again 10 times with the buffers and the absorbance was read at 510 nm. Total anthocyanin content was determined by the following equation:

(1) anthocyanin content  (%)=[(Abs pH  1.0−Abs pH  4.5)×484.82×DF(24825×Wt)],

where 484.82 is the molecular mass of cyaniding-3-glicoside chloride, 24825 is molar absorptivity at 510 nm in pH = 1, DF is the dilution factor, and Wt is the sample weight.

### 2.3. Preparation of Medicinal and Placebo Capsules

The concentrated extract was mixed with tribasic calcium phosphate powder, granulated, and dried, and then its anthocyanin content was quantified. The medicinal capsules were filled with the mixed granules so that each contained 500 mg of dried granules equivalent to 45 ± 2 mg of total anthocyanin. The placebo capsules with shape, color, and size similar to medicinal ones were filled only with dried granulated tribasic calcium phosphate.

### 2.4. Patient Selection

All hyperlipidemic adult patients who met the following inclusion criteria were recruited in the study: (1) age ≥ 18 years, (2) serum levels of total cholesterol 200–300 mg/dL and/or triglyceride (TG) 150–199 mg/dL and/or LDL-C 130 130–190 mg/dL, (3) being non-smokers, (4) free of diseases affecting serum lipids (e.g., diabetes, thyroid disorders, and pancreatitis), (5) not using drugs or supplements affecting serum lipids (e.g., statins, fibrate derivatives, estrogens, progestins, beta-blockers, thiazide diuretics, and fish oil) within the last 3 months, (6) free of liver or kidney disease, and (7) nonpregnant, nonlactating women.

### 2.5. Study Design and Interventions

This was a randomized, double-blind, placebo-controlled clinical trial performed in Isfahan cardiovascular research center affiliated to Isfahan University of Medical Sciences, Isfahan, Iran, from September 2012 to January 2013. Informed consent was obtained from all participants and the study protocol was approved by the ethical committee (Research Ethics Committee of Isfahan Cardiovascular Research Center, Isfahan, Iran). Patients with inclusion criteria were randomly and equally assigned to either the study drug (*Vaccinium*) or placebo groups. Randomization was performed using random number table. Before intervention, the demographic characteristics (including BMI) and fasting serum levels of total cholesterol, TG, LDL-C, HDL-C, hs-CRP, and malondialdehyde (MDA) were recorded for all patients. Also, to detect any possible side effects of the drug on the liver and kidney, the serum levels of ALT, AST, BUN, and creatinine were obtained. The patients of drug and placebo groups used two medicinal and placebo capsules, respectively, per day with food for 4 weeks. All patients were instructed to maintain their usual diet and physical activity and report any adverse effect during the study. The patients compliance was evaluated by counting their capsules at the end of use and their results were included in data analysis if they used more than 80% of their capsules. At the end of 4 weeks, all above-mentioned parameters were again measured and compared to baseline values. All participants, the physician, and laboratory personnel were blind to intervention type.

### 2.6. Statistical Analysis

SPSS 20.0 software was used for statistical analysis of resulting data. Kolmogorov-Smirnov test was performed to assess normal distribution of continuous data. Because of normal distribution of all continuous data, Student's *t*-test was used for comparisons. Paired-samples *t*-test was performed for comparison of measurements at the beginning and end of intervention within each group. General linear model (multivariate) analysis was used for comparing the mean changes of each parameter form baseline between drug (*Vaccinium*) and placebo groups. Chi-square test was performed for comparison of gender distribution in two groups. The level of significance was considered as *P* < 0.05.

## 3. Results

**Figure 1:**
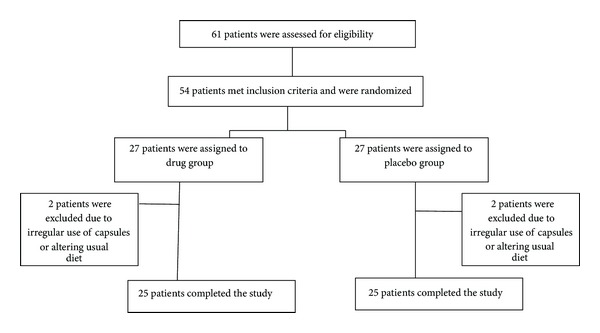
Flowchart of patient's enrollment in the study.

During the study, a total of 61 hyperlipidemic subjects were screened, with 54 found to be eligible according to the inclusion criteria; however, 2 subjects were excluded from each group during the trial because of either irregular use of capsules or altering their usual diet. Therefore, statistical analyses were performed for a total of 50 subjects (25 in each group) who fully completed the trial (Figure 1).

Table 1 shows baseline demographic and clinical characteristics of study patients. As shown, all subjects were matched regarding baseline values.

Table 2 presents changes of tested parameters from baseline after 4 weeks of intervention in *Vaccinium* and placebo groups and comparison of them. As shown, *Vaccinium* significantly reduced total cholesterol, LDL-C, TG, and MDA compared to placebo but had not any statistically significant effect on other tested parameters.

Table 3 shows the effects of *Vaccinium* and placebo on laboratory markers of liver and kidney function after 4 weeks of use. No significant changes were detected in these values during the study. Also, no complication or adverse effect was reported by patients of both groups.

## 4. Discussion

In the present study, we investigated the effects of fruit concentrated extract of *Vaccinium arctostaphylos* on serum lipid profile and serum levels of hs-CRP and MDA in hyperlipidemic adult patients. Only one previous study, published at the time of this writing, exists for the effects of *Vaccinium arctostaphylos* on lipid profile of hyperlipidemic patients [23]; however, several studies have been conducted for some other species of the genus Vaccinium. Our study showed cholesterol-, LDL-C-, and triglyceride-lowering effects of the berries of *Vaccinium arctostaphylos*, but no significant effect was observed on HDL-C. Similarly, in the above-mentioned recently published study conducted by Kianbakht et al., the use of hydroalcoholic extract of *V. arctostaphylos* (one 350 mg capsule every 8 hours) for 2 months, compared to placebo, significantly lowered the blood levels of total cholesterol (*P* < 0.001), TG (*P* = 0.002), and LDL-C (*P* = 0.002) [23]; however, in contrast to our result, the extract significantly increased the blood HDL-C levels (*P* < 0.001). This difference could be due to higher dose of consumed extract and longer duration of this study. The lipid-lowering effects of some other species of Vaccinium have been demonstrated in several animal studies [24–26] and clinical trials [27, 28]. In the study of Lee et al., the use of cranberry (*Vaccinium macrocarpon*) extract with the daily dose of 1500 mg for 12 weeks by patients with type 2 diabetes caused significant reduction in serum levels of LDL-C and total cholesterol and total cholesterol/HDL cholesterol ratio [28]. In contrast to our results, in a study of patients with features of metabolic syndrome, daily consumption of 400 g fresh bilberries (*Vaccinium myrtillus*) for 8 weeks had not any significant effect on serum lipids [29]. In another study performed by Basu et al., the use of cranberry (*Vaccinium macrocarpon*) juice for 8 weeks did not significantly affect lipid profile in patients with metabolic syndrome [30]. The observed lipid-lowering effects of *V. arctostaphylos* in the present study may be due to its anthocyanin content. Also, water-soluble fibers in the berries could be responsible for reduction of serum cholesterol through binding to bile salts in the intestine leading to inhibition of their enterohepatic cycle [31]. It is noteworthy that the administration of our tested extract for a longer period of time might be more effective at lowering the serum lipids.

In the present research, *V. arctostaphylos* had not any significant effect on the serum level of hs-CRP in hyperlipidemic subjects. In the study of Kolehmainen et al., daily intake of 400 g fresh bilberries (*Vaccinium myrtillus*) for 8 weeks significantly reduced serum hs-CRP and some other inflammatory markers [29]. Also, in the study of Karlsen et al., supplementation with 330 mL bilberry juice/day for 4 weeks resulted in significant decrease in plasma concentrations of CRP [32]. Conversely, others have shown no significant effects [33–35]. Considering a slight decrease in hs-CRP by *V. arctostaphylos* (in contrast to placebo) observed in our study, the use of higher doses of this extract for longer periods may have more significant effect on this inflammatory marker.

In our research, *V. arctostaphylos *fruits extract showed significant effect in reduction of MDA as a marker of oxidative stress compared to placebo (*P* = 0.013). Consistently, in the study of Basu et al., daily use of cranberry juice for 8 weeks caused a significant decrease in both ox-LDL and MDA versus placebo treatment (−33% versus −17% and −50% versus +7%, resp.) [30]; however, in this study, the baseline MDA values were higher than those of our subjects and the duration of intervention was longer. Our results show antioxidant activity of the fruit extracts of *V. arctostaphylos*. This suggests that it can be considered as a nutritional supplement for prevention of atherosclerosis in high-risk patients including hyperlipidemic subjects as increasing evidence indicates inhibition of LDL oxidation through attenuation of oxidative stress is beneficial in preventing the development of atherosclerosis [12]. The reduction of plasma MDA concentrations in response to *V. arctostaphylos* is likely to be attributable to its polyphenolic compounds as they exert a potent antioxidant activity [36, 37].

In conclusion, daily consumption of the fruit extract of *Vaccinium arctostaphylos* significantly reduces the serum levels of total cholesterol, LDL-C, and triglyceride (TG) and oxidative stress in hyperlipidemic patients. Therefore, this extract could be considered as a potential agent for treatment of dyslipidemia and prevention of atherosclerosis development. However, more studies with higher doses and longer periods of time are mandatory.

## Figures and Tables

**Table 1:** Baseline demographic and clinical characteristics of study patients The values are presented as mean ± SD.

Parameter (unit)	Vaccinium (*n* = 25)	Placebo (*n* = 25)	*P* value
Age (years)	48.08 ± 16.39	46.36 ± 16.59	0.714
Gender (%male)	40.0	40.0	0.723
BMI (kg/m^2^)	25.40 ± 1.75	25.21 ± 2.01	0.720
Total cholesterol (mg/dL)	226.48 ± 32.09	220.20 ± 45.76	0.577
LDL-C (mg/dL)	132.80 ± 23.76	121.08 ± 32.06	0.149
TG (mg/dL)	226.20 ± 96.99	191.36 ± 56.54	0.129
HDL-C (mg/dL)	45.76 ± 9.73	46.56 ± 10.52	0.781
hs-CRP (mg/L)	2.53 ± 2.33	2.80 ± 2.35	0.737
MDA (µmol/L)	0.738 ± 0.266	0.707 ± 0.212	0.653

**Table 2:** The effects of interventions on tested parameters after 4 weeks in study patients. The values are presented as mean ± SD.

Parameter (unit)	Vaccinium (*n* = 25)	Placebo (*n* = 25)	*P*-value
BMI (kg/m^2^)			
Baseline	25.40 ± 1.75	25.21 ± 2.01	0.062
End	25.06 ± 1.60	25.31 ± 2.07	
Change	−0.33 ± 0.45	0.10 ± 0.45	
Total cholesterol (mg/dL)			
Baseline	226.48 ± 32.09	220.20 ± 45.76	<0.001
End	192.04 ± 28.81	223.52 ± 42.31	
Change	−34.44 ± 22.44	3.32 ± 15.47	
LDL-C (mg/dL)			
Baseline	132.80 ± 23.76	121.08 ± 32.06	0.004
End	121.36 ± 27.46	124.36 ± 30.29	
Change	−11.44 ± 3.28	3.28 ± 16.04	
TG (mg/dL)			
Baseline	226.20 ± 96.99	191.36 ± 56.54	<0.001
End	156.56 ± 46.76	198.56 ± 63.30	
Change	−69.64 ± 76.86	7.20 ± 27.51	
HDL-C (mg/dL)			
Baseline	45.76 ± 9.73	46.56 ± 10.52	0.631
End	45.60 ± 9.72	45.68 ± 9.72	
Change	−0.16 ± 6.36	−0.88 ± 3.85	
hs-CRP (mg/L)			
Baseline	2.53 ± 2.33	2.80 ± 2.35	0.190
End	1.99 ± 1.60	3.04 ± 2.34	
Change	−0.54 ± 2.83	0.23 ± 0.49	
MDA (µmol/L)			
Baseline	0.738 ± 0.266	0.707 ± 0.212	0.013
End	0.648 ± 0.16	0.769 ± 0.26	
Change	−0.09 ± 0.23	0.06 ± 0.18	

**Table 3:** The effects of interventions on the liver and kidney function tests after 4 weeks. The values are presented as mean ± SD.

Parameter (unit)	Vaccinium (*n* = 25)			Placebo (*n* = 25)		
	Baseline	Week 4	*P*-value	Baseline	Week 4	*P*-value
ALT (U/L)	22.48 ± 10.88	20.88 ± 11.51	0.193	24.72 ± 9.12	24.24 ± 8.18	0.643
AST (U/L)	21.60 ± 7.77	20.60 ± 7.94	0.246	23.20 ± 7.48	23.68 ± 8.48	0.580
BUN (mg/dL)	14.28 ± 3.27	13.96 ± 4.60	0.616	14.60 ± 4.41	13.36 ± 4.76	0.075
Creatinine (mg/dL)	0.96 ± 0.15	0.92 ± 0.14	0.166	0.96 ± 0.23	0.93 ± 0.22	0.513
